# Protective Role of a *TMPRSS2* Variant on Severe COVID-19 Outcome in Young Males and Elderly Women

**DOI:** 10.3390/genes12040596

**Published:** 2021-04-19

**Authors:** Maria Monticelli, Bruno Hay Mele, Elisa Benetti, Chiara Fallerini, Margherita Baldassarri, Simone Furini, Elisa Frullanti, Francesca Mari, GEN-COVID Multicenter Study, Giuseppina Andreotti, Maria Vittoria Cubellis, Alessandra Renieri

**Affiliations:** 1 Department of Biology, Università Federico II, 80126 Napoli, Italy; maria.monticelli@unina.it (M.M.); bruno.haymele@szn.it (B.H.M.); 2 Integrative Marine Ecology Department, Stazione Zoologica Anton Dohrn, Villa Comunale, 80121 Napoli, Italy; 3 Med Biotech Hub and Competence Center, Department of Medical Biotechnologies, University of Siena, 53100 Siena, Italy; elisa.benetti@dbm.unisi.it (E.B.); fallerini2@unisi.it (C.F.); margherita.baldassarri@dbm.unisi.it (M.B.); simone.furini@unisi.it (S.F.); elisa.frullanti@dbm.unisi.it (E.F.); francesca.mari@unisi.it (F.M.); alessandra.renieri@unisi.it (A.R.); 4 Medical Genetics, Department of Medical Biotechnologies, University of Siena, 53100 Siena, Italy; 5 Genetica Medica, Azienda Ospedaliero-Universitaria Senese, 53100 Siena, Italy; 6 Istituto di Chimica Biomolecolare—CNR, 80078 Pozzuoli, Italy

**Keywords:** COVID-19, *TMPRSS2*, V197M, missense mutation, Whole-Exome Sequencing (WES)

## Abstract

The protease encoded by the *TMPRSS2* gene facilitates viral infections and has been implicated in the pathogenesis of SARS-CoV-2. We analyzed the *TMPRSS2* sequence and correlated the protein variants with the clinical features of a cohort of 1177 patients affected by COVID-19 in Italy. Nine relatively common variants (allele frequency > 0.01) and six missense variants which may affect the protease activity according to PolyPhen-2 in HumVar-trained mode were identified. Among them, p.V197M (p.Val197Met) (rs12329760) emerges as a common variant that has a deleterious effect on the protease and a protective effect on the patients. Its role appears particularly relevant in two subgroups of patients—young males and elderly women—and among those affected by co-morbidities, where the variant frequency is higher among individuals who were mildly affected by the disease and did not need hospitalization or oxygen therapy than among those more severely affected, who required oxygen therapy, ventilation or intubation. This study provides useful information for the identification of patients at risk of developing a severe form of COVID-19, and encourages the usage of drugs affecting the expression of *TMPRSS2* or inhibiting protein activity.

## 1. Introduction

The gene *TMPRSS2* encodes a plasma membrane-anchored serine protease (TMPS2_HUMAN) that activates several endogenous substrates such as pro-hepatocyte growth factor [1], PAR-2 [2], matriptase/ST14 [3], and ACE2 [4]. In addition to this, it is essential for the pathogenesis of several human viruses, coronavirus 229E [5], Middle East respiratory syndrome coronavirus [6], influenza A viruses [7], and parainfluenza viruses [8].

The protein is composed of a small cytoplasmic region (aa 1–121), a transmembrane helix (aa 122–142), and an extracellular region (aa 143–529). In turn, the last one is composed of three domains, an LDL-receptor-like domain (aa 149–186), a SRCR-like domain (aa 187–279), and a peptidase domain (aa 293–526).

*TMPRSS2* is expressed in the prostate glands predominantly, but also in type II pneumocytes in the lung, nasal goblet secretory cells, and small intestine [9,10,11,12]. It is regulated by androgenic hormones in vivo [1].

Genetic determinants of susceptibility and/or severity of COVID-19 have been sought in *TMPRSS2* in predictive [13,14,15,16] as well as in Whole-Exome Sequencing (WES) studies [17,18]. An analysis extended to large cohorts confirms the protective role of a common polymorphism p.V197M (rs12329760) in *TMPRSS2* and, in particular, its effect in young males and elderly women.

## 2. Materials and Methods

Whole-Exome Sequencing (WES) data derived from the GEN-COVID Multicenter Study [19] were analyzed. Odds ratio (OR) for different contingency tables were calculated using unconditional maximum likelihood estimation (Wald’s method); confidence intervals (CI) at 95% were calculated using the normal approximation. Independence was tested using a Chi-squared test. All calculations were performed within the R environment for statistical computing, using the OR function from the epitools package. R scripts are provided in Supplementary file S1.

The sequence of human TMPS2_HUMAN was obtained from the UniProt database [20]. Structural templates were searched using the SWISS-MODEL server [21] and a model of the region spanning aa 187 to 526 was built using the structure of human hepsin [22]. A protein model was visualized using PyMol [23]. The solvent accessibility and the effect of mutations on protein stability were calculated for variants occurring in the region spanning aa 187 to 526 using SDM [24].

PolyPhen-2 [25], which is a program that can graduate the severity of missense variants [26], was used to identify mutations with a high impact on TMPS2_HUMAN.

## 3. Results

**Figure 1:**
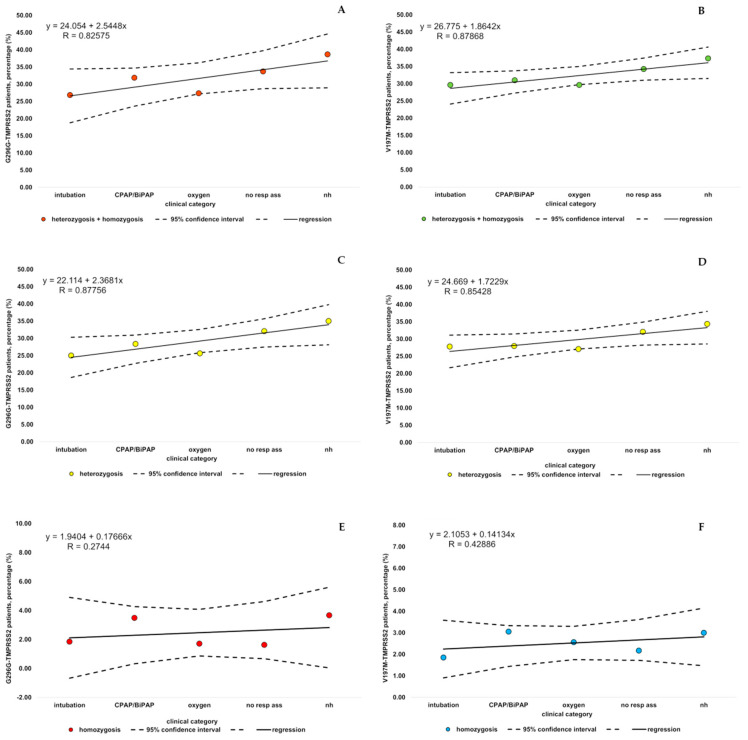
Prevalence of p.G296G or p.V197M in COVID-19 patients subdivided by different clinical outcomes. The percentages of carriers (**A**,**B**), heterozygous (**C**,**D**) and homozygous (**E**,**F**) individuals for p.G296G (**A**,**C**,**E**) or p.V197M (**B**,**D**,**F**) in each clinical category, i.e., patients who needed intubation, ventilation (CPAP/BiPAP), or oxygen therapy, patients who did not need oxygen therapy (no resp support) or were not hospitalized (nh), are reported. The 95% confidence interval around the regression line is calculated as the product of the regression standard error and the value of the 0.975-th quantile of a t distribution with 3 degrees of freedom.

We analyzed the WES data of 1177 patients affected by COVID-19 and selected according to the following inclusion criteria: (i) endotracheal intubation; (ii) CPAP/BiPAP ventilation; (iii) oxygen therapy; (iv) hospitalized without oxygen support; and (v) not hospitalized. In this cohort, we identified 52 variants in *TMPRSS2*, nine of which are relatively common (allele frequency > 0.01). *TMPRSS2* encodes two isoforms that differ for 37 aa at the amino-terminus and both can activate respiratory viruses [27]. In this paper, the numbering of the longest isoform is used to identify variants in coding regions. The frequencies of two common mutations, one missense and one synonymous, p.V197M (rs12329760) and p.G296G (rs2298659), correlate with COVID-19 severity (Figure 1 and Table 1).

The percentage of carriers in severely (intubation + CPAP/BiPAP + oxygen) or mildly (no resp support + nh) affected patients differs significantly and is higher in the latter group (p.V197M *p* = 0.0289, OR = 0.7601, LC = 0.5941, HC = 0.9724; p.G296G *p* = 0.0039, OR = 0.6947, LC = 0.5422 HC = 0.8899; Table 1).

Patients were divided in two categories according to the severity of the disease: mild (including not hospitalized and no respiratory assistance required) and severe (including oxygen therapy, ventilation, or intubation required).

Both SNPs are covered by the GWAS study of Severe COVID-19 with Respiratory Failure and are significantly associated with susceptibility (p.V197M *p* = 0.0153 OR = 0.8497, p.G296G *p* = 0.0462 OR = 0.8730) [28].

**Figure 2:**
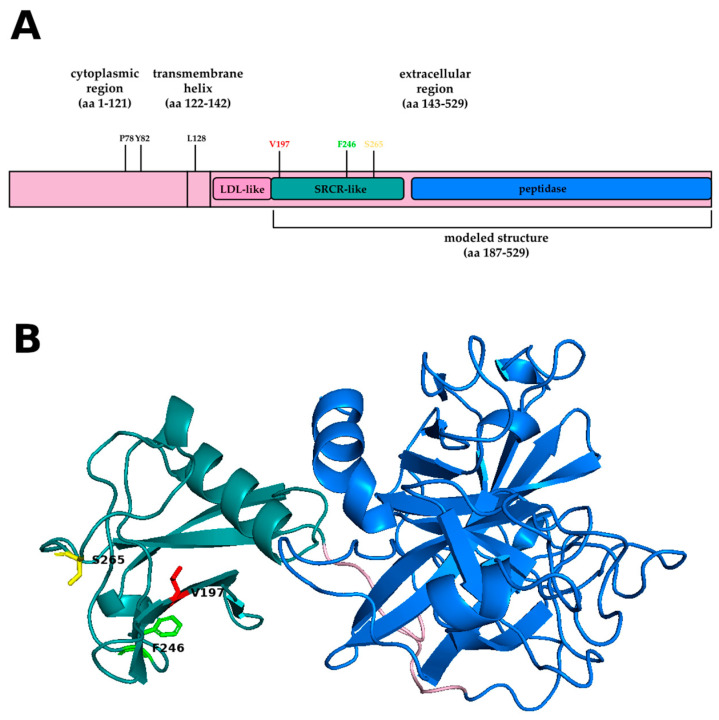
TMPRSS2 structure. A linear representation of TMPRSS2 is shown in panel (**A**). The probably and possibly damaging mutations identified by the HumVar-trained model in wAnnovar are highlighted. The model of the region spanning from aa 187 to 526 is shown as a cartoon in panel (**B**). The SRCR-like domain is in deep teal cyan, peptidase domain is in marine blue, and the linker is in light pink. V197 (red), F246 (green), and S265 (yellow) belong to the SRCR-like domain.

Several lines of evidence support the hypothesis that p.V197M affects the stability and/or the function of the protease. It is predicted to be deleterious by SIFT [29] and PolyPhen-2 [25], in both predictive modes, HumDiv and HumVar (Supplementary file S2).

p.V197M does not occur in the catalytic domain but in the SRCR domain that is likely needed for protein-protein interaction [30,31]. The region spanning aa 187 to 526 can be built by homology modeling. The side chain of Valine197 is buried (Figure 2), and solvent accessibility (%) 15.4 and its substitution by Methionine affects the stability of the protein (pseudo∆∆G= −2.00 kcal/mol).

On the other side, the synonymous variant p.G296G, which corresponds to NM_001135099:exon9:c.C888T, is not annotated as an eQTL and does not fall at the exon-intron junction. We hypothesize that the missense mutation is protective and is often associated in cis with the synonymous variant, which has little effect per se.

The frequency of the other common variants does not correlate with the disease severity (Supplementary file S3). This is expected since most of them are synonymous or intron variants. The only missense mutation is p.G8V that is predicted to be benign by SIFT [29] and PolyPhen-2 [25] in both predictive modes, HumDiv and HumVar (Supplementary file S2).

It has been observed and it is commonly accepted that age, sex, and co-morbidities influence the severity of COVID-19. We divided patients according to sex and age (below and above median values) and the severity of the disease. The number of carriers of p.V197M (heterozygous + homozygous individuals) among mild and severe cases differs significantly in two sub-cohorts, those of young males (*p* = 0.0200, OR = 0.5804, LC = 0.3663, HC = 0.9197) and elderly women (*p* = 0.0347, OR = 0.5346, LC = 0.2977, HC = 0.9601) (Table 2). To check whether slightly altering the age frame may flip the *p*-value to non-significant, we tested different ages and found that, provided that the boundary between young and old patients falls between 52 and 66 years for males and between 54 and 70 years for females, the protective effect remains significant for young males and elderly women (Supplementary Figure S1).

We restricted the two groups of patients and analyzed young males and elderly women who were affected by co-morbidities such as diabetes and/or hypertension and/or obesity or other diseases (young males affected by co-morbidities *p* = 0.0057, OR = 0.2969, LC = 0.1254, HC = 0.7027; elderly women affected by co-morbidities *p* = 0.0391, OR = 0.4667, LC = 0.2262, HC = 0.9627) (Table 3). The results hold significance when we consider the first quartile of the male or the last quartile of the female population (very young males in Q1 affected by co-morbidities *p* = 0.0343, OR = 0.2121, LC = 0.0485, HC = 0.9281; very elderly women in Q4 affected by co-morbidities *p* = 0.0090, OR = 0.2455, LC = 0.0812, HC = 0.7424) (Table 4).

Data in the two sub-cohorts of elderly males and young women are shown in Supplementary file S4. The subdivision of patients was carried out starting from the complete data set reporting the age, sex, and co-morbidities of individual patients. It is provided in Supplementary file S5.

Patients were divided according to sex and age (above and below median age) and the severity of the disease.

Patients with co-morbidities were divided according to sex and age (above and below median age) and the severity of the disease.

Patients were divided according to sex and age (quartiles) and the severity of the disease.

It is not surprising that the effects of a deleterious mutation in *TMPRSS2* are seen in both sexes because the expression of the gene is only slightly higher in males [13]. The difference in the times of life can be explained because androgens and estrogens have opposite effects on gene expression, as proved by the data collected from the Expression Atlas [32] (Supplementary file S6). Young males who do not carry p.V197M are at risk because of high testosterone levels. Particularly relevant is the risk of wild-type elderly women who are not protected by estrogens. Androgenic hormones decline with age less rapidly than estradiol after menopause and this effect might explain the risk in elderly females and the protective role of the variant.

Other rare missense mutations are found in the Italian cohort in heterozygosity (Supplementary file S2). We will discuss the germline mutations that can affect the protein. A few fall in the region spanning aa 187 to 526 that can be modeled by homology, thus precluding a comparative analysis based on structural effects. PolyPhen-2 [25] uses sequence conservation to predict deleterious effects and two databases for training and testing predictions. The HumDiv model is trained using Mendelian disease variants vs. divergence from close mammalian homologs of human proteins (≥95% sequence identity). HumVar is trained using all human variants associated with some disease (except cancer mutations) or loss of activity/function vs. common (minor allele frequency > 1%) human polymorphism with no reported association with a disease of other effects.

The HumVar-trained model, which is best suited for distinguishing mutations with drastic effects, predicts that only one rare variant, p.L128P (rs147711290), has a strong effect (probably damaging, D, supplementary file S2) on the protein, perhaps because it occurs in the transmembrane anchor where proline destabilizes the helix [33]. p.L128P, which is relatively frequent only among Ashkenazy Jews (0.2%), was found in the Italian cohort in a single patient on oxygen therapy. The HumVar-trained model predicts that p.S265I and p.F246I (rs150554820), which occur on the surface of SRCR-like domain (aa 187–279) (Figure 2), p.P78L (rs138651919) and p. Y82D (rs201679623), which are located in the poorly characterized cytoplasmic region, have a moderate effect (possibly damaging, P, supplementary file S2) that is not sufficient to protect the patients. The effects of p.S265I and p.F246I (rs150554820) were calculated using the model shown in Figure 2. Both mutations affect relatively buried amino acids, S265 and F246 (Solvent accessibility (%) 11.5 and 1.2, respectively), but have a very mild effect on protein stability (S265I and F246I pseudo∆∆G = −0.29 kcal/mol and −0.07 kcal/mol, respectively).

These variants are not enriched in the mildly affected patients but were observed in severely affected ones too, 5 and 6, respectively.

## 4. Discussion and Conclusions

From our analysis, based on the WES of a large cohort of Italian patients, a variant in *TMPRSS2* emerges as a predictive factor to identify patients at risk of a severe course of COVID-19.

The allele frequency of p.V197M in our study is 0.18, in line with what was already reported (0.17 [13]). This value is well below the one reported in GnomAD for Eastern Asians (0.38), Finns (0.37), and Africans (0.29). Among Non-Finnish Europeans, the allele frequency of p.V197M is 0.23 with an apparent gradient from North to South, Northern Sweden 0.29, Estonia 0.31 (Genetic variation in the Estonian population), UK 0.21 (UK 10K study—Twins), and Spain 0.17 (Medical Genome Project healthy controls from Spanish population). It is highly suggestive correlating the low frequency of V197M with the high impact that the first wave of the epidemics had in Italy.

Other missense mutations are not enriched in the mildly affected patients, possibly because their effect on the protein is weak.

The protein encoded by *TMPRSS2* belongs to a family of membrane proteases, some of which promote SARS-CoV-2 infection [4,5,11]. No common deleterious missense mutations are found in these genes but for a variant in *TMPRSS4*, p.P413L, which is frequent among Latinos and mixed Americans (0.1259) but not among Europeans (0.002).

The fact that a missense mutation with a destabilizing effect on the protein product, such as p.V197M, influences the course of COVID-19 implicitly suggests that *TMPRSS2* can be targeted for therapies either reducing its expression or inhibiting its protein product.

Sexual hormones can be used for therapeutic purposes [34,35]. Quite interestingly, and in line with our results, Seeland et al. reported that: “their retrospective study of hormone therapy in female COVID-19 patients shows that the fatality risk for women > 50 years receiving estradiol therapy (user group) is reduced by more than 50%; the OR was 0.33, 95% CI (0.18, 0.62) and the hazard ratio (HR) was 0.29, 95% CI (0.11,0.76). For younger, pre-menopausal women (15–49 years), the risk of COVID-19 fatality is the same irrespective of estradiol treatment, probably because of higher endogenous estradiol levels” [36].

To obtain more targeted effects, specific inhibitors at the protein level could be considered since they could have a protective effect analogous to that exerted by the mutation. Preliminary clinical data concerning Camostat [37,38] and Nafamostat [39,40] have been published. Their identification was the result of reposition which is a useful approach to reduce the time and costs of drug development [41] and has been largely employed during the emergency posed by COVID-19 [42,43].

In vitro Camostat and Nafamostat bind and inhibit TMPS2_HUMAN with great affinity, IC50 6.2 nM and 0.27 nM, respectively, but are not specific [44] and are not devoid of side effects in the patients [45]. In silico docking experiments have been carried out to find other inhibitors of TMPS2_HUMAN [46], but in vitro validation of the hits has not yet been carried out.

The effect of V197M on TMPS2_HUMAN was predicted by several authors [13,14,15,16,17,18]. WES analysis conducted on a large cohort of patients proves that the variant has indeed a statistically significant protective role in COVID-19. We do hope that our study will not only help to identify at risk patients, especially among elderly women, but also encourage the development of drugs for their treatment.

During the revision of this paper, we became aware that an independent research group proved the protective role of *TMPRSS2* variants in COVID-19 in the general population [47].

## Figures and Tables

**Table 1:** Contingency tables of *TMPRSS2* mutations in the two categories of patients.

(A) p.G296G			
	Mild	Severe	Marginal_Rows
Wild-type	306 (63%)	490 (71%)	796
G296G	178 (37%)	198 (29%)	376
*marginal_cols*	484	688	1172
**(** **B) p.V197M**			
	**Mild**	**Severe**	**Marginal_Rows**
Wild-type	313 (64%)	482 (70%)	795
V197M	176 (36%)	206 (30%)	382
*marginal_cols*	489	688	1177

**Table 2:** Contingency tables of V197M TMPRSS2 in young males and elderly women.

(A) Male, Young (Age ≤ 60)			
	Mild	Severe	Marginal_Rows
Wild-type	102 (63%)	140 (75%)	242
Val197Met	59 (37%)	47 (25%)	106
*marginal_cols*	161	187	348
**(B) Female, Elderly (Age ≥ 58)**			
	**Mild**	**Severe**	**Marginal_Rows**
Wild-type	37 (57%)	131 (71%)	168
Val197Met	28 (43%)	53 (29%)	81
*marginal_cols*	65	184	249

**Table 3:** Contingency tables of V197M TMPRSS2 in young males and elderly women affected by co-morbidities.

(A) Male with Co-Morbidities, Young (Age ≤ 60)			
	Mild	Severe	Marginal_Rows
Wild-type	16 (50%)	65 (77%)	81
Val197Met	16 (50%)	19 (23%)	35
*marginal_cols*	32	84	116
**(B) Female with Co-Morbidities, Elderly (Age ≥ 58)**			
	**Mild**	**Severe**	**Marginal_Rows**
Wild-type	21 (54%)	105 (71%)	126
Val197Met	18 (46%)	42 (29%)	60
*marginal_cols*	39	147	186

**Table 4:** Contingency tables of V197M TMPRSS2 in very young males and very elderly women affected by co-morbidities.

(A) Male, Very Young (Age ≤ 50)			
	Mild	Severe	Marginal_Rows
Wild-type	7 (39%)	12 (75%)	19
Val197Met	11 (61%)	4 (25%)	15
*marginal_cols*	18	16	34
**(B) Female, Very Elderly (Age ≥ 71)**			
	**Mild**	**Severe**	**Marginal_Rows**
Wild-type	6 (37.5%)	66 (71%)	72
Val197Met	10 (62.5%)	27 (29%)	37
*marginal_cols*	16	93	109

## Data Availability

The data and samples referenced here are housed in the GEN-COVID Patient Registry and the GEN-COVID Biobank and are available for consultation. You may contact Alessandra Renieri (e-mail: alessandra.renieri@unisi.it).

## Supplementary Materials

The following are available online at https://www.mdpi.com/article/10.3390/genes12040596/s1, Supplementary Tables S1: R scripts; Supplementary Tables S2: *TMPRSS2* variants in COVID-19 patients’ cohort; Supplementary Tables S3: contingency tables of *TMPRSS2* common variants with no correlation; Supplementary Tables S4: contingency tables of old males and young women; Supplementary Tables S5: complete data set reporting the age, sex, and co-morbidities of individual patients; Supplementary Tables S6: differential expression data for *TMPRSS2* from Expression Atlas. Supplementary Figure S1: influence of the boundary age on the statistical significance of p.V197M protective effect.

## Appendix A

Collaborators of GEN-COVID Multicenter Study

Sergio Daga (Med Biotech Hub and Competence Center, Department of Medical Biotechnologies, University of Siena, Italy; Medical Genetics, Department of Medical Biotechnologies, University of Siena, Italy), Francesca Fava (Med Biotech Hub and Competence Center, Department of Medical Biotechnologies, University of Siena, Italy; Medical Genetics, Department of Medical Biotechnologies, University of Siena, Italy; Genetica Medica, Azienda Ospedaliero-Universitaria Senese, Italy), Floriana Valentino (Med Biotech Hub and Competence Center, Department of Medical Biotechnologies, University of Siena, Italy; Medical Genetics, Department of Medical Biotechnologies, University of Siena, Italy), Gabriella Doddato (Med Biotech Hub and Competence Center, Department of Medical Biotechnologies, University of Siena, Italy; Medical Genetics, Department of Medical Biotechnologies, University of Siena, Italy), Annarita Giliberti (Med Biotech Hub and Competence Center, Department of Medical Biotechnologies, University of Siena, Italy; Medical Genetics, Department of Medical Biotechnologies, University of Siena, Italy), Rossella Tita (Genetica Medica, Azienda Ospedaliero-Universitaria Senese, Italy), Sara Amitrano (Genetica Medica, Azienda Ospedaliero-Universitaria Senese, Italy), Mirella Bruttini (Med Biotech Hub and Competence Center, Department of Medical Biotechnologies, University of Siena, Italy; Medical Genetics, Department of Medical Biotechnologies, University of Siena, Italy; Genetica Medica, Azienda Ospedaliero-Universitaria Senese, Italy), Susanna Croci (Med Biotech Hub and Competence Center, Department of Medical Biotechnologies, University of Siena, Italy; Medical Genetics, Department of Medical Biotechnologies, University of Siena, Italy), Ilaria Meloni (Med Biotech Hub and Competence Center, Department of Medical Biotechnologies, University of Siena, Italy; Medical Genetics, Department of Medical Biotechnologies, University of Siena, Italy), Anna Maria Pinto (Genetica Medica, Azienda Ospedaliero-Universitaria Senese, Italy), Maria Antonietta Mencarelli (Genetica Medica, Azienda Ospedaliero-Universitaria Senese, Italy), Caterina Lo Rizzo (Genetica Medica, Azienda Ospedaliero-Universitaria Senese, Italy), Francesca Montagnani (Med Biotech Hub and Competence Center, Department of Medical Biotechnologies, University of Siena, Italy; Dept of Specialized and Internal Medicine, Tropical and Infectious Diseases Unit, Azienda Ospedaliera Universitaria Senese, Siena, Italy), Laura Di Sarno (Med Biotech Hub and Competence Center, Department of Medical Biotechnologies, University of Siena, Italy; Medical Genetics, Department of Medical Biotechnologies, University of Siena, Italy), Andrea Tommasi (Med Biotech Hub and Competence Center, Department of Medical Biotechnologies, University of Siena, Italy; Medical Genetics, Department of Medical Biotechnologies, University of Siena, Italy; Genetica Medica, Azienda Ospedaliero-Universitaria Senese, Italy), Maria Palmieri (Med Biotech Hub and Competence Center, Department of Medical Biotechnologies, University of Siena, Italy; Medical Genetics, Department of Medical Biotechnologies, University of Siena, Italy), Miriam Lucia Carriero (Med Biotech Hub and Competence Center, Department of Medical Biotechnologies, University of Siena, Italy; Medical Genetics, Department of Medical Biotechnologies, University of Siena, Italy), Diana Alaverdian (Med Biotech Hub and Competence Center, Department of Medical Biotechnologies, University of Siena, Italy; Medical Genetics, Department of Medical Biotechnologies, University of Siena, Italy), Giada Beligni (Med Biotech Hub and Competence Center, Department of Medical Biotechnologies, University of Siena, Italy; Medical Genetics, Department of Medical Biotechnologies, University of Siena, Italy), Nicola Iuso (Med Biotech Hub and Competence Center, Department of Medical Biotechnologies, University of Siena, Italy; Medical Genetics, Department of Medical Biotechnologies, University of Siena, Italy), Gabriele Inchingolo (Med Biotech Hub and Competence Center, Department of Medical Biotechnologies, University of Siena, Italy; Medical Genetics, Department of Medical Biotechnologies, University of Siena, Italy), Massimiliano Fabbiani (Dept of Specialized and Internal Medicine, Tropical and Infectious Diseases Unit, Azienda Ospedaliera Universitaria Senese, Siena, Italy), Barbara Rossetti (Dept of Specialized and Internal Medicine, Tropical and Infectious Diseases Unit, Azienda Ospedaliera Universitaria Senese, Siena, Italy), Giacomo Zanelli (Med Biotech Hub and Competence Center, Department of Medical Biotechnologies, University of Siena, Siena, Italy; Dept of Specialized and Internal Medicine, Tropical and Infectious Diseases Unit, Azienda Ospedaliera Universitaria Senese, Siena, Italy), Elena Bargagli (Unit of Respiratory Diseases and Lung Transplantation, Department of Internal and Specialist Medicine, University of Siena), Laura Bergantini (Unit of Respiratory Diseases and Lung Transplantation, Department of Internal and Specialist Medicine, University of Siena), Miriana D’Alessandro (Unit of Respiratory Diseases and Lung Transplantation, Department of Internal and Specialist Medicine, University of Siena), Paolo Cameli (Unit of Respiratory Diseases and Lung Transplantation, Department of Internal and Specialist Medicine, University of Siena), David Bennet (Unit of Respiratory Diseases and Lung Transplantation, Department of Internal and Specialist Medicine, University of Siena), Federico Anedda (Dept of Emergency and Urgency, Medicine, Surgery and Neurosciences, Unit of Intensive Care Medicine, Siena University Hospital, Italy), Simona Marcantonio (Dept of Emergency and Urgency, Medicine, Surgery and Neurosciences, Unit of Intensive Care Medicine, Siena University Hospital, Italy), Sabino Scolletta (Dept of Emergency and Urgency, Medicine, Surgery and Neurosciences, Unit of Intensive Care Medicine, Siena University Hospital, Italy), Federico Franchi (Dept of Emergency and Urgency, Medicine, Surgery and Neurosciences, Unit of Intensive Care Medicine, Siena University Hospital, Italy), Maria Antonietta Mazzei (Department of Medical, Surgical and Neurosciences and Radiological Sciences, Unit of Diagnostic Imaging, University of Siena), Susanna Guerrini (Department of Medical, Surgical and Neurosciences and Radiological Sciences, Unit of Diagnostic Imaging, University of Siena), Edoardo Conticini (Rheumatology Unit, Department of Medicine, Surgery and Neurosciences, University of Siena, Policlinico Le Scotte, Italy), Luca Cantarini (Rheumatology Unit, Department of Medicine, Surgery and Neurosciences, University of Siena, Policlinico Le Scotte, Italy), Bruno Frediani (Rheumatology Unit, Department of Medicine, Surgery and Neurosciences, University of Siena, Policlinico Le Scotte, Italy), Danilo Tacconi (Department of Specialized and Internal Medicine, Infectious Diseases Unit, San Donato Hospital Arezzo, Italy), Chiara Spertilli (Department of Specialized and Internal Medicine, Infectious Diseases Unit, San Donato Hospital Arezzo, Italy), Marco Feri (Dept of Emergency, Anesthesia Unit, San Donato Hospital, Arezzo, Italy), Alice Donati (Dept of Emergency, Anesthesia Unit, San Donato Hospital, Arezzo, Italy), Raffaele Scala (Department of Specialized and Internal Medicine, Pneumology Unit and UTIP, San Donato Hospital, Arezzo, Italy), Luca Guidelli (Department of Specialized and Internal Medicine, Pneumology Unit and UTIP, San Donato Hospital, Arezzo, Italy), Genni Spargi (Department of Emergency, Anesthesia Unit, Misericordia Hospital, Grosseto, Italy), Marta Corridi (Department of Emergency, Anesthesia Unit, Misericordia Hospital, Grosseto, Italy), Cesira Nencioni (Department of Specialized and Internal Medicine, Infectious Diseases Unit, Misericordia Hospital, Grosseto, Italy), Leonardo Croci (Department of Specialized and Internal Medicine, Infectious Diseases Unit, Misericordia Hospital, Grosseto, Italy), Gian Piero Caldarelli (Laboratory Medicine Department, Misericordia Hospital, Grosseto, Italy), Maurizio Spagnesi (Department of Preventive Medicine, Azienda USL Toscana Sud Est, Italy), Paolo Piacentini (Department of Preventive Medicine, Azienda USL Toscana Sud Est, Italy), Maria Bandini (Department of Preventive Medicine, Azienda USL Toscana Sud Est, Italy), Elena Desanctis (Department of Preventive Medicine, Azienda USL Toscana Sud Est, Italy), Silvia Cappelli (Department of Preventive Medicine, Azienda USL Toscana Sud Est, Italy), Anna Canaccini (Territorial Scientific Technician Department, Azienda USL Toscana Sud Est, Italy), Agnese Verzuri (Territorial Scientific Technician Department, Azienda USL Toscana Sud Est, Italy), Valentina Anemoli (Territorial Scientific Technician Department, Azienda USL Toscana Sud Est, Italy), Agostino Ognibene (Laboratory Medicine Department, San Donato Hospital, Arezzo, Italy), Alessandro Pancrazi (Laboratory Medicine Department, San Donato Hospital, Arezzo, Italy), Maria Lorusso (Laboratory Medicine Department, San Donato Hospital, Arezzo, Italy), Massimo Vaghi (Chirurgia Vascolare, Ospedale Maggiore di Crema, Italy), Antonella D’Arminio Monforte (Department of Health Sciences, Clinic of Infectious Diseases, ASST Santi Paolo e Carlo, University of Milan, Italy), Esther Merlini (Department of Health Sciences, Clinic of Infectious Diseases, ASST Santi Paolo e Carlo, University of Milan, Italy), Federica Gaia Miraglia (Department of Health Sciences, Clinic of Infectious Diseases, ASST Santi Paolo e Carlo, University of Milan, Italy), Mario U. Mondelli (Division of Infectious Diseases and Immunology, Fondazione IRCCS Policlinico San Matteo, Pavia, Italy; Department of Internal Medicine and Therapeutics, University of Pavia, Italy), Raffaele Bruno (Division of Infectious Diseases and Immunology, Fondazione IRCCS Policlinico San Matteo, Pavia, Italy; Department of Internal Medicine and Therapeutics, University of Pavia, Italy), Vecchia Marco (Division of Infectious Diseases and Immunology, Fondazione IRCCS Policlinico San Matteo, Pavia, Italy), Stefania Mantovani (Division of Infectious Diseases and Immunology, Fondazione IRCCS Policlinico San Matteo, Pavia, Italy), Serena Ludovisi (Division of Infectious Diseases and Immunology, Fondazione IRCCS Policlinico San Matteo, Pavia, Italy; Department of Internal Medicine and Therapeutics, University of Pavia, Italy), Massimo Girardi (Department of Anesthesia and In-tensive Care, University of Modena and Reggio Emilia, Modena, Italy), Sophie Venturelli (Department of An-esthesia and Intensive Care, University of Modena and Reggio Emilia, Modena, Italy), Marco Sita (Department of Anesthesia and Intensive Care, University of Modena and Reggio Emilia, Modena, Italy), Andrea Cossarizza (Department of Medical and Surgical Sciences for Children and Adults, University of Modena and Reggio Emi-lia, Modena, Italy), Andrea Antinori (HIV/AIDS Department, National Institute for Infectious Diseases, IRCCS, Lazzaro Spallanzani, Rome, Italy), Alessandra Vergori (HIV/AIDS Department, National Institute for Infectious Diseases, IRCCS, Lazzaro Spallanzani, Rome, Italy), Arianna Emiliozzi (Med Biotech Hub and Competence Center, Department of Medical Biotechnologies, University of Siena, Siena, Italy; Dept of Specialized and Internal Medicine, Tropical and Infectious Diseases Unit, Azienda Ospedaliera Universitaria Senese, Siena, Italy; HIV/AIDS Department, National Institute for Infectious Diseases, IRCCS, Lazzaro Spal-lanzani, Rome, Italy), Stefano Rusconi (III Infectious Diseases Unit, ASST-FBF-Sacco, Milan, Italy; Department of Biomedical and Clinical Sciences Luigi Sacco, University of Milan, Milan, Italy), Matteo Siano (Department of Biomedical and Clinical Sciences Luigi Sacco, University of Milan, Milan, Italy), Arianna Gabrieli (Depart-ment of Biomedical and Clinical Sciences Luigi Sacco, University of Milan, Milan, Italy), Agostino Riva (III Infectious Diseases Unit, ASST-FBF-Sacco, Milan, Italy; Department of Biomedical and Clinical Sciences Luigi Sacco, University of Milan, Milan, Italy), Daniela Francisci (Infectious Diseases Clinic, Department of Medicine, Azienda Ospedaliera di Perugia and University of Peru-gia, Santa Maria Hospital, Perugia, Italy; Infectious Diseases Clinic, “Santa Maria” Hospital, University of Perugia, Perugia, Italy), Elisabetta Schiarol (Infectious Diseases Clinic, Department of Medicine, Azienda Ospedaliera di Perugia and University of Peru-gia, Santa Maria Hospital, Perugia, Italy), Francesco Paciosi (Infectious Diseases Clinic, Department of Medi-cine, Azienda Ospedaliera di Perugia and University of Perugia, Santa Maria Hospital, Perugia, Italy), Pier Giorgio Scotton (Department of Infectious Diseases, Treviso Hospital, Local Health Unit 2 Marca Trevigiana, Treviso, Italy), Francesca Andretta (Department of Infectious Diseases, Treviso Hospital, Local Health Unit 2 Marca Trevigiana, Treviso, Italy), Sandro Panese (Clinical Infectious Diseases, Mestre Hospital, Venezia, Italy), Renzo Scaggiante (Infectious Diseases Clinic, ULSS1, Belluno, Italy), Francesca Gatti (Infectious Diseases Clinic, ULSS1, Belluno, Italy), Saverio Giuseppe Parisi (Department of Molecular Medicine, University of Padova, Italy), Francesco Castelli (Department of Infectious and Tropical Diseases, University of Brescia and ASST Spedali Civili Hospital, Brescia, Italy), Eugenia Quiros-Roldan (Department of Infectious and Tropical Diseases, University of Brescia and ASST Spedali Civili Hospital, Brescia, Italy), Melania degli Antoni (Department of Infectious and Tropical Diseases, University of Brescia and ASST Spedali Civili Hospi-tal, Brescia, Italy), Isabella Zanella (Department of Molecular and Translational Medicine, University of Bre-scia, Italy; Clinical Chemistry Laboratory, Cytogenetics and Molecular Genetics Section, Diagnostic Depart-ment, ASST Spedali Civili di Brescia, Italy), Matteo Della Monica (Medical Genetics and Laboratory of Medi-cal Genetics Unit, A.O.R.N. “Antonio Cardarelli”, Naples, Italy), Carmelo Piscopo (Medical Genetics and La-boratory of Medical Genetics Unit, A.O.R.N. “Antonio Cardarelli”, Naples, Italy), Mario Capasso (Department of Molecular Medicine and Medical Biotechnology, University of Naples Federico II, Naples, Italy; CEINGE Biotecnologie Avanzate, Naples, Italy; IRCCS SDN, Naples, Italy), Roberta Russo (Department of Molecular Medicine and Medical Biotechnology, University of Naples Federico II, Naples, Italy; CEINGE Biotecnologie Avanzate, Naples, Italy), Immacolata Andolfo (Department of Molecular Medicine and Medical Biotechnology, University of Naples Federico II, Naples, Italy; CEINGE Biotecnologie Avanzate, Naples, Italy), Achille Iolascon (Department of Molecular Medicine and Medical Biotechnology, University of Naples Federico II, Naples, Italy; CEINGE Biotecnologie Avanzate, Naples, Italy), Giuseppe Fiorentino (Unit of Respiratory Physiopathology, AORN dei Colli, Monaldi Hospital, Naples, Italy), Massimo Carella (Division of Medical Genetics, Fondazione IRCCS Casa Sollievo della Sofferenza Hospital, San Giovanni Rotondo, Italy), Marco Castori (Division of Medical Genetics, Fondazione IRCCS Casa Sollievo della Sofferenza Hospital, San Giovanni Rotondo, Italy), Giuseppe Merla (Division of Medical Genetics, Fondazione IRCCS Casa Sollievo della Sofferenza Hospital, San Giovanni Rotondo, Italy), Gabriella Maria Squeo (Division of Medical Genetics, Fondazione IRCCS Casa Sollievo della Sofferenza Hospital, San Giovanni Rotondo, Italy), Filippo Aucella (Department of Medical Sciences, Fondazione IRCCS Casa Sollievo della Sofferenza Hospital, San Giovanni Rotondo, Italy), Pamela Raggi (Clinical Trial Office, Fondazione IRCCS Casa Sollievo della Sofferenza Hospital, San Giovanni Rotondo, Italy), Carmen Marciano (Clinical Trial Office, Fondazione IRCCS Casa Sollievo della Sofferenza Hospital, San Giovanni Rotondo, Italy), Rita Perna (Clinical Trial Office, Fondazione IRCCS Casa Sollievo della Sofferenza Hospital, San Giovanni Rotondo, Italy), Matteo Bassetti (Department of Health Sciences, University of Genova, Genova, Italy; Infectious Diseases Clinic, Policlinico San Martino Hospital, IRCCS for Cancer Research Genova, Italy), Antonio Di Biagio (Infectious Diseases Clinic, Policlinico San Martino Hospital, IRCCS for Cancer Research Genova, Italy), Maurizio Sanguinetti (Microbiology, Fondazione Policlinico Universitario Agostino Gemelli IRCCS, Catholic University of Medicine, Rome, Italy; Department of Laboratory Sciences and Infectious Diseases, Fondazione Policlinico Universitario A. Gemelli IRCCS, Rome, Italy), Luca Masucci (Microbiology, Fondazione Policlinico Universitario Agostino Gemelli IRCCS, Catholic University of Medicine, Rome, Italy; Department of Laboratory Sciences and Infectious Diseases, Fondazione Policlinico Universitario A. Gemelli IRCCS, Rome, Italy), Serafina Valente (Department of Cardiovascular Diseases, University of Siena, Siena, Italy), Marco Mandalà (Otolaryngology Unit, University of Siena, Italy), Alessia Giorli (Otolaryngology Unit, University of Siena, Italy), Lorenzo Salerni (Otolaryngol-ogy Unit, University of Siena, Italy), Patrizia Zucchi (Department of Internal Medicine, ASST Valtellina e Alto Lario, Sondrio, Italy), Pierpaolo Parravicini (Department of Internal Medicine, ASST Valtellina e Alto Lario, Sondrio, Italy), Elisabetta Menatti (Study Coordinator Oncologia Medica e Ufficio Flussi, Sondrio, Italy), Stefano Baratti (Department of Infectious and Tropical Diseases, University of Padova, Padova, Italy), Tullio Trotta (First Aid Department, Luigi Curto Hospital, Polla, Salerno, Italy), Ferdinando Giannattasio (First Aid Department, Luigi Curto Hospital, Polla, Salerno, Italy), Gabriella Coiro (First Aid Department, Luigi Curto Hospital, Polla, Salerno, Italy), Fabio Lena (Local Health Unit-Pharmaceutical Department of Grosseto, Toscana Sud Est Local Health Unit, Grosseto, Italy), Domenico A. Coviello (U.O.C. Laboratorio di Genetica Umana, IRCCS Istituto G. Gaslini, Genova, Italy), Cristina Mussini (Infectious Diseases Clinics, University of Modena and Reggio Emilia, Modena, Italy), Giancarlo Bosio (Department of Respiratory Diseases, Azienda Ospedaliera di Cremona, Cremona, Italy), Enrico Martinelli (Department of Respiratory Diseases, Azienda Ospedaliera di Cremona, Cremona, Italy), Sandro Mancarella (U.O.C. Medicina: ASST Nord Milano, Ospedale Bassini, Cinisello Balsamo (MI), Italy), Luisa Tavecchia (U.O.C. Medicina: ASST Nord Milano, Ospedale Bassini, Cinisello Balsamo (MI), Italy), Lia Crotti (Istituto Auxologico Italiano, IRCCS, Department of Cardiovascular, Neural and Metabolic Sciences, San Luca Hospital, Milan, Italy; Department of Medicine and Surgery, University of Milano-Bicocca, Milan, Italy; Istituto Auxologico Italiano, IRCCS, Center for Cardiac Arrhythmias of Genetic Origin, Milan, Italy; Istituto Auxologico Italiano, IRCCS, Laboratory of Cardiovascular Genetics, Milan, Italy; Member of the European Reference Network for Rare, Low Prevalence and Complex Diseases of the Heart-ERN GUARD-Heart), Gianfranco Parati (Istituto Auxologico Italiano, IRCCS, Department of Cardiovascular, Neural and Metabolic Sciences, San Luca Hospital, Milan, Italy; Department of Medicine and Surgery, University of Milano-Bicocca, Milan, Italy), Nicola Picchiotti (University of Siena, DIISM-SAILAB, Siena, Italy; Department of Mathematics, University of Pavia, Pavia, Italy), Marco Gori (University of Siena, DIISM-SAILAB, Siena, Italy; University Cote d’Azur, Inria, CNRS, I3S, Maasai), Chiara Gabbi (Independent Medical Scientist, Milan, Italy), Maurizio Sanarico (Independent Data Scientist, Milan, Italy), Stefano Ceri (Department of Electronics, Information and Bioengineering (DEIB), Politecnico di Milano, Milano, Italy), Pietro Pinoli (Department of Electronics, Information and Bioengineering (DEIB), Politecnico di Milano, Mila-no, Italy), Francesco Raimondi (Scuola Normale Superiore, Pisa, Italy), Filippo Biscarini (CNR-Consiglio Nazionale delle Ricerche, Istituto di Biologia e Biotecnologia Agraria (IBBA), Milano, Italy), Alessandra Stella (CNR-Consiglio Nazionale delle Ricerche, Istituto di Biologia e Biotecnologia Agraria (IBBA), Milano, Italy), Marco Rizzi (Unit of Infectious Diseases, ASST Papa Giovanni XXIII Hospital, Bergamo, Italy), Franco Maggiolo (Unit of Infectious Diseases, ASST Papa Giovanni XXIII Hospital, Bergamo, Italy), Diego Ripamonti (Unit of Infectious Diseases, ASST Papa Giovanni XXIII Hospital, Bergamo, Italy), Claudia Suardi (Fondazione per la ricerca Ospedale di Bergamo, Bergamo, Italy), Tiziana Bachetti (Direzione Scientifica, Istituti Clinici Scientifici Maugeri IRCCS, Pavia, Italy), Maria Teresa La Rovere (Istituti Clinici Scientifici Maugeri IRCCS, Department of Cardiology, Institute of Montescano, Pavia, Italy), Simona Sarzi-Braga (Istituti Clinici Scientifici Maugeri, IRCCS, Department of Cardiac Rehabilitation, Institute of Tradate (VA), Italy), Maurizio Bussotti (Cardiac Rehabilitation Unit, Fondazione Salvatore Maugeri, IRCCS, Scientific Institute of Milan, Mi-lan, Italy), Mattia Bergomi (Veos Digital, Milan, Italy), Katia Capitani (Med Biotech Hub and Competence Center, Department of Medical Biotechnologies, University of Siena, Siena, Italy; Core Research Laboratory, ISPRO, Florence, Italy), Kristina Zguro (Med Biotech Hub and Competence Center, Department of Medical Bi-otechnologies, University of Siena, Siena, Italy) and Simona Dei (Health Management, Azienda USL Toscana Sudest, Tuscany, Italy).
