# Supplementary material for: Protective Role of a TMPRSS2 Variant on Severe COVID-19 Outcome in Young Males and Elderly Women
Source: Genes (Basel). 2021 Apr 19;12(4):596. doi: 10.3390/genes12040596 (PMC8073081; doi:10.3390/genes12040596)
Supplement: Supplementary file 1 [file genes-12-00596-s001.zip › Supplementary materials/S6 extra_tab_ATLAS.pdf]

genes

MDPI

**Supplementary table 6. Differential expression data for *TMPRSS2*.**

Data from Expression Atlas for *TMPRSS2* in Homo sapiens. Data related to sexual hormones were selected over 212 experiments.

| Experiment accession | Comparison | log_2 fold change | Adjusted *p*-value |
|---|---|---|---|
| **E-MTAB-5537** | 'estrogen receptor alpha shRNA' vs 'scrambled shRNA' | -6.7 | 5.52E-37 |
| **E-MTAB-7294** | 'Enzalutamide, 1 micromolar' vs 'vehicle' at '48 hour' | -3 | 3.37E-99 |
| **E-MTAB-7294** | 'Enzalutamide, 1 micromolar' vs 'vehicle' at '24 hour' | -2.6 | 9.90E-130 |
| **E-GEOD-48403** | 'after androgen ablation therapy' vs 'before androgen ablation therapy' | -2.2 | 2.01E-13 |
| **E-GEOD-11428** | 'abl; dihydrotestosterone; 4 hour' vs 'abl; vehicle' | 1.5 | 3.35E-06 |
| **E-GEOD-11791** | '17beta-estradiol; 100 nanomolar; wild type' vs 'ethanol; wild type' | -1.5 | 0.000118 |
| **E-GEOD-57935** | 'estradiol; 1 nanomolar' vs 'vehicle' in 'control' at '24 hour' | -1.4 | 7.99E-05 |
| **E-GEOD-42619** | 'E2; 10 nanomolar' vs 'control; E2 ' | -1.1 | 0.027774 |
