# Supplementary figures and images for: Protective Role of a TMPRSS2 Variant on Severe COVID-19 Outcome in Young Males and Elderly Women
Source: Genes (Basel). 2021 Apr 19;12(4):596. doi: 10.3390/genes12040596 (PMC8073081; doi:10.3390/genes12040596)

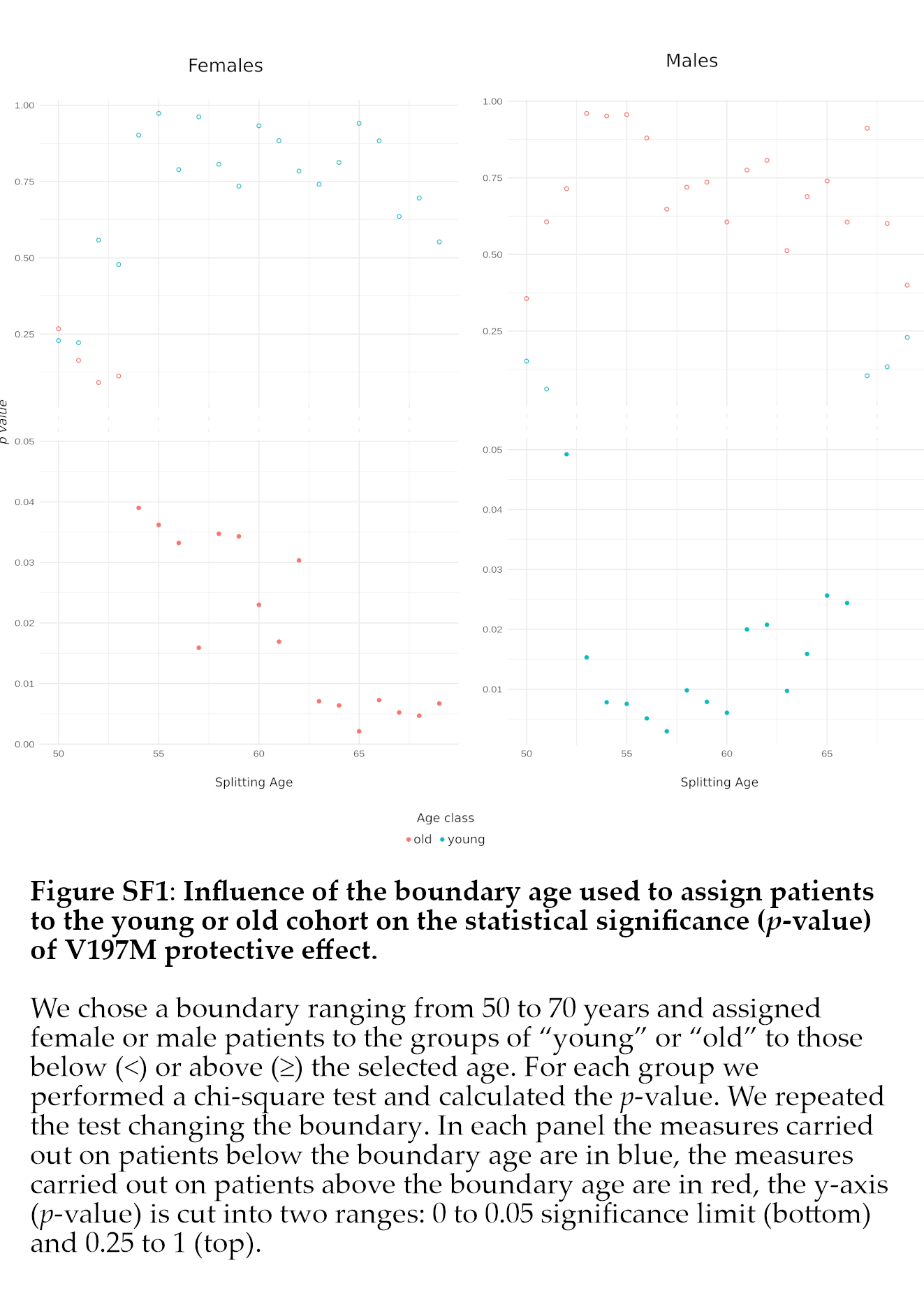

Supplement: Supplementary file 1 [file genes-12-00596-s001.zip › Supplementary materials/SF1.png]
